# The Role of Serum Dickkopf1 and CKAP4 Levels in Diagnosing Colorectal Cancer and Measuring the Disease Severity: A Prospective Study

**DOI:** 10.3390/medicina60060933

**Published:** 2024-06-03

**Authors:** Esra Dişçi, Rıfat Peksöz, Esra Laloğlu, Mehmet İlhan Yıldırgan, Yavuz Albayrak, Mehmet Akif Şirin, Enes Ağırman, Sabri Selçuk Atamanalp

**Affiliations:** 1 Department of General Surgery, Faculty of Medicine, Atatürk University, Erzurum 25240, Turkey; esra.disci16@gmail.com (E.D.); rifat-peksoz@hotmail.com (R.P.); ilhanyildirgan@gmail.com (M.İ.Y.);; 2 Department Medical Biochemistry, Faculty of Medicine, Atatürk University, Erzurum 25240, Turkey; 3 Department of General Surgery, Erzurum City Hospital, Erzurum 25240, Turkey

**Keywords:** DKK1, CKAP4, colorectal cancer, biomarker, disease severity

## Abstract

*Background and Objective:* Colorectal cancer (CRC) is among the most common types of cancer. Although the disease is treatable in its early stages, five-year survival falls below 20% in the later stages. CEA and CA19-9 are tumor markers used in the diagnosis and follow-up of the disease in clinical practice; however, their diagnostic effectiveness is insufficient. Therefore, the identification of biomarkers that can be easily studied from serum and can diagnose CRC and determine its severity is highly important. In this context, dickkopf1 (DKK1) and cytoskeleton-associated protein 4 (CKAP4) are both promising biomarkers. *Materials and Methods:* Serum DKK1 and CKAP4 levels were measured in 55 patients with CRC and 40 healthy controls. The patients with CRC were divided into groups based on pathological stages and histological differentiation. The serum levels of both proteins in patients with CRC were measured preoperatively and 10 and 30 days postoperatively. *Results:* Serum DKK1 and CKAP4 were significantly higher in the CRC group than in the healthy controls (*p* < 0.05). Serum levels of both proteins rose in line with the disease stage and grade but decreased following surgical resection. A positive correlation was observed between tumor diameter and protein blood levels. The diagnostic efficacy of DKK1 and CKAP4 in CRC (approximately 95%) was higher than that of markers such as CEA and CA19-9. *Conclusions:* The DKK1 and CKAP4 serum values of patients with CRC are promising biomarkers. They can potentially be used in CRC management, namely, in the diagnosis and treatment of tumor response access and in tumor aggressiveness prediction.

## 1. Introduction

Colorectal cancer (CRC) is one of the most common types of cancer worldwide. CRC is the third most common type of cancer in Türkiye and the second most fatal type of cancer in the USA [1,2]. This cancer typically does not develop suddenly but slowly turns into cancer in the course of time. Due to this slow and progressive characteristic, the majority of tumors can be diagnosed in the early stages, and the disease can be treated via endoscopic or surgical excision of the lesions [3]. When the tumor is diagnosed in a more advanced phase, the stage of the disease is crucial in terms of treatment and survival. The five-year survival rate for local early cancers is 90%; however, it is only 13% in patients with distant metastasis [4]. Although progress has been made in its treatment, 20–45% of patients who undergo curative resection subsequently develop distant metastasis or experience tumor recurrence [5]. The disease must be closely monitored since recurrence may be observed, even following radical treatment. It is therefore important to identify the disease early or to know the post-treatment recurrence status to overcome the disease.

Computed tomography (CT) and endoscopic procedures are currently applied to detect colorectal polyps and cancers. CT colonography is another alternative for detecting colorectal cancer when endoscopic procedures cannot be applied. In addition, CT scanning is currently used to determine tumor stage the disease severity. However, despite their high diagnostic values, these methods also have significant disadvantages, such as high cost, an invasive character, and time-consuming procedures. While less-costly and non-invasive methods are available, such as the fecal occult blood test, their sensitivity and specificity are low or not at the desired level [3,6]. Simple, non-invasive methods with a high diagnostic value are therefore needed. Ideally, these markers should provide information about the stage of the disease based on their serum levels, and it is also important to use them in postoperative follow-up. Several biomarkers are used for the early diagnosis, treatment, staging, and prognosis of colorectal cancer. Carcinoembryonic antigen (CEA) and carbohydrate antigen 19-9 (CA19-9) are biomarkers routinely employed in the diagnosis and follow-up of colonic cancers in clinical practice [7]. However, the levels of these markers can also increase with other diseases. Their value in diagnosis and follow-up is therefore limited. In this context, there is a need for new markers.

Dickkopf1 (DKK1) and cytoskeleton-associated membrane protein 4 (CKAP4) are proteins that have been the subject of numerous studies in recent years. CKAP acts as the DKK1 receptor, and these proteins form the DKK1/CKAP4 signaling pathway, which is involved in both normal and cancer cell proliferation. The ability to investigate these as biomarkers in serum may be promising in terms of diagnosing CRC and managing the disease [5,8,9]. Researchers have also investigated the potential use of antibodies to both proteins in the treatment of cancer [9].

This study aimed to investigate the serum levels of the proteins DKK1 and CKAP4, a subject of considerable research in the context of cancer in recent years with respect to patients with CRC. This study presents promising results in revealing the association between these proteins, disease stage, histological tumor grade, and changes in serum biomarker levels post-surgery.

## 2. Materials and Methods

### 2.1. Patients and Methods

Ethical approval for this single-center prospective study was granted by the institutional review board (Ataturk University Faculty of Medicine ethical committee, no. B.30.2.ATA.0.01.00, dated 24 June 2021). Informed consent was obtained from all patients. This study was conducted in accordance with the 1964 Declaration of Helsinki and its later amendments.

In total, 105 patients with colorectal adenocarcinoma operated on at Atatürk University Research Hospital between July 2021 and March 2023 were included in our study. Fifty patients who did not meet the inclusion criteria or whose blood results were unavailable for any reason were excluded from the study. To minimize the effects on serum DKK1/CKAP4 levels, patients with a primary tumor other than colorectal cancer (n = 12), a history of chemotherapy or radiotherapy except for CRC (n = 6), more than one comorbid disease, significant systemic disease (n = 8), and whose serums could not be transferred to the laboratory (n = 24) were also excluded from the study. The 40 healthy controls should be selected from patients with no chronic diseases who have recently had a normal screening colonoscopy. As a result, our study included 50 patients and 40 healthy controls.

Blood placed into biochemistry tubes for the measurement of serum DKK1 and CKAP4 levels before surgery and on the postoperative days 10 and 30 was centrifuged and stored at −80 °C until use. Blood was collected once from the healthy control group. Tumor markers such as CEA and CA19-9 were measured among laboratory values routinely obtained before the surgery (Table 1).

Serum DKK1 and CKAP were compared (1) between the pre- and postoperative periods; (2) according to pathological TNM staging; and (3) according to histological grades (Table 2, Table 3 and Table 4).

Tumor staging was based on postoperative pathology reports and was conducted in line with the TNM staging system for CRC. TNM staging was performed in line with the Eighth American Joint Committee on Cancer. Tumors were classified histologically as well- (Grade 1), moderately (Grade 2), or poorly (Grade 3) differentiated according to the predominant cell type [10,11]. DKK1 and CKAP4 correlations were evaluated. Correlations were evaluated between tumor diameter and the two proteins. ROC curves were produced to determine the capacity of laboratory values to differentiate between healthy controls and CRC. The data were retrieved from the patients’ files and the hospital’s electronic software system.

### 2.2. Blood Specimens

Before initiating any drug treatment for the patients and control groups included in this study, blood samples obtained for routine biochemistry tests were taken for 10–20 min for clotting. After the tube was kept in an upright position, it was centrifuged at +4 °C at 4000 rpm for 15 min. The serum samples obtained were aliquoted and placed in the deep freezer at −80 °C. CKAP4 and DKK1 levels in serum samples were studied via the ELISA method.

### 2.3. Analyte Assay Techniques

Serum obtained from whole blood samples collected at admission were analyzed via enzyme-linked immunosorbent assay (ELISA) using the Human CKAP4 ELISA Kit (BT LAB, Cat. No. E4664Hu, Shanghai, China) and the Human DKK1 ELISA Kit (BT LAB, Cat. No. E0630Hu, China) according to the manufacturer’s instructions. The inter-assay and intra-assay coefficients of variance given by the manufacturer are <10% and <8%, respectively. Briefly, the samples and standards were added to wells pre-coated with human CKAP4 and DKK1 antibody. The CKAP4 and DKK1 present in the samples were bound by the antibodies coating the wells. Biotinylated human CKAP4 and DKK1 antibody was then added to bind to the bound CKAP4 and DKK1, followed by streptavidin–horseradish peroxidase (HRP) to bind to the biotinylated CKAP4 and DKK1 antibody. After incubation, the unbound streptavidin–HRP was washed away. A substrate solution was added, and the color developed in proportion to the amount of human CKAP4 and DKK1 in the well. The reaction was terminated by adding acidic stop solution, and absorbance was measured at 450 nm. CKAP4 and DKK1 concentrations were determined by comparing the optical density in the sample wells with the standard curve. The results were expressed as ng/L and ng/mL, respectively. CEA and CA 19-9 tests of the patients were studied via the chemiluminescence method on UniCel DxI 800 (Beckman Coulter Diagnostics, Brea, CA, USA) immunoassay device.

### 2.4. Statistical Analyses

Data recording and analysis was conducted using “SPSS 20.0 for Windows” (SPSS Inc., Chicago, IL, USA) software. Descriptive data were expressed as number and % for categoric variables and median (minimum and maximum) or mean ± standard deviation for numeric variables. The normality of distribution was evaluated using visual (histograms, probability plots) and analytical methods (Kolmogorov–Smirnov/Shapiro–Wilk test). The study groups were compared using one-way analysis of variance (ANOVA), and the significance of differences between groups was evaluated using the post hoc Bonferroni test. Moreover, due to the non-parametric distribution of serum CEA and CA 19-9 levels, the Mann–Whitney U test was used to calculate significance. The correlation analyses were performed using Pearson’s correlation. The ROC curve, which is an expression of a technique’s predictive power, was used to determine sensitivity, specificity, and cut-off values for serum CKAP4, DKK-1, CEA, and CA 19-9 levels. At this point, the ROC method will put forward that an optical density (OD) value from ELISA with the highest Youden Index (J) is an optimal cutoff value to differentiate cancer and healthy serum sample. A *p*-value of <0.05 was considered to be statistically significant.

## 3. Results

The study was conducted with 55 patients admitted to Atatürk University Medical Faculty general surgery clinic with the diagnosis of CRC between July 2021 and March 2023. The control group consisted of 40 healthy volunteers or volunteers with no unknown important diseases.

While 32 (58.2%) patients with CRC were male, 23 (41.8%) were female. In the control group, while 21 (52.5%) were male, 19 (47.5%) were female (*p* = 0.582, chi-square test). The mean age was 63.27 ± 9.7 (36–81) years in the CRC group and 58.12 ± 7.8 (30–71) in the control group (*p* = 0.076). No significant difference was determined between the control group and patients with CRC regarding gender and age.

A comorbid disease was present in 51% of the CRC group patients, while no important comorbidity was present in the control group. The tumor was in the right side of the colon in 12 patients, on the left in 22, and in the rectum in 21. Neoadjuvant therapy was administered to 18 (32.7%) patients. Thirty (54.5%) patients were operated on laparoscopically and twenty-five (45.5%) using open surgery. Regarding postoperative pathological evaluation of the patients’ colectomy specimens (the TNM staging system), 11 patients were categorized as stage I, 18 as stage II, 21 as stage III, and 5 as stage IV. In terms of degrees of tumor differentiation, 4 patients were categorized as Grade 1, 43 as Grade 2, and 8 as Grade 3 (Table 1).

How serum DKK1 and CKAP4 levels changed according to the severity of the disease, as well as with the reduction of the tumor burden following surgical resection, were examined.

The levels of both proteins increased in line with the stage of the disease. Although no statistically significant difference was observed for either protein between stages 3 and 4 (*p* > 0.05), all the other stages differed significantly from one another (*p* < 0.05) (Table 2).

The serum levels of both markers rose in line with histological grades. Although no significant difference was observed for either protein between grades 2 and 3 (*p* > 0.05), all the other grades differed significantly (*p* < 0.05) (Table 3).

Serum CKAP4 and DKK1 levels were significantly higher in the patients with CRC compared to the control group. Blood levels in the patients with CRC were higher in the pre-surgical resection period but decreased on the 10th day postoperatively and reached their lowest on the 30th day (*p* < 0.05) (Table 4).

Significant, powerful, positive correlations were observed between DKK1 and CKAP4 (r = 0.825, *p* < 0.001). Significant positive correlations were also determined between tumor diameter and DKK1 and CKAP4 levels (r = 0.307, *p* = 0.027 for DKK1 and r = 0.356, *p* = 0.01 for CKAP4) (Table 5).

Sensitivity values for CEA, CA19-9, DKK1, and CKAP4 in diagnosing CRC in the preoperative period were 77%, 75%, 94%, and 98%, respectively, while specificity values were 74%, 87%, 97%, and 96% (Table 6). Although markers such as CEA and CA 19-9 routinely used in clinical practice are of high diagnostic efficacy, DKK1 and CKAP4 proteins exhibited greater diagnostic effectiveness (approximately 95%).

## 4. Discussion

DKK1 is a secretory protein and a member of the DKK family. It inhibits the Wnt signaling pathway by binding to the Wnt coreceptor low-density lipoprotein receptor-related protein 6 (LRP6). It binds to LRP6 and CKAP4 with similar affinities [12]. It is also overexpressed in cancer cells and promotes migration, proliferation, and invasion. Levels of DKK1 frequently increase in different cancers [13]. However, DKK1 has been reported to exhibit a suppressant character in cancers such as colon cancer, breast cancer, and renal cell carcinoma. Therefore, whether DKK1 has a suppressive or oncogenic effect on cancer cells is therefore controversial [14]. DKK1 has been shown to act as a proto-oncogene in patients with hepatocellular carcinoma (HCC). High DKK1 expression in patients with HCC indicates its ability to be a biomarker or molecular target [9,15]. These proteins’ tissue expression levels have generally been investigated, and few studies have considered their serum levels. Significantly higher serum DKK1 levels have been determined in patients with lung and esophageal cancer than in healthy control groups. Serum DKK1 levels also decreased significantly following surgical resection [16]. DKK1 levels in serum have also been investigated in patients with colon cancer, with higher levels being observed compared to healthy individuals. Studies have also suggested that preoperative serum DKK1 levels appear to represent a potential marker of tumor invasion and recurrence in stage II–III colon cancer [17].

Inconsistent with several previous studies, serum DKK1 levels were significantly higher in the patients with colon cancer in our study compared to in the control group. Serum protein levels also increased in line with the disease stage and grade. The significant decrease in serum DKK1 levels following surgical resection revealed a positive correlation between the tumor burden and protein levels.

CKAP4, the second protein on the DKK1/CKAP4 signaling pathway, is a receptor for DKK1 localized in the plasma membrane. DKK1 activates the PI3K-AKT pathway by binding to CKAP4 and thus stimulates cell proliferation. Simultaneous expression of the two proteins is linked to poor prognosis in esophageal, lung, and pancreatic cancers. Experimental studies have shown that anti-CKAP4 antibody inhibits tumor formation in these cancers. CKAP4 may therefore constitute a molecular target for the treatment of these cancers [9]. It has also recently been identified as a serological marker of several tumors and may also represent a biological marker for both the diagnosis and molecular targeting of tumors [18]. High serum CKAP4 levels have been determined in patients with pancreatic cancer and pancreatic-tumor-bearing xenografted mice. Serum levels decreased in the postoperative period, becoming barely detectable. Kimura H. et al. reported that anti-CKAP4 monoclonal antibodies inhibited the proliferation and migration of pancreatic cancer cells by suppressing the binding of DKK1 to CKAP4—in other words, by inhibiting the AKT axis. The application of these antibodies to mice given pancreatic cancer cells was also seen to prolong their life spans [19]. As can also be seen from the above-mentioned information, the use of DKK1 and CKAP4 antibodies in the treatment process has been mentioned in various studies, and the use of these antibodies in the treatment of this disease is a positive development for the future [13,18,19].

The protein’s degree of expression affects survival. However, studies regarding to what extent the degree of expression of CKAP4 affects survival are inconsistent. High CKAP4 expression has a positive impact on overall and disease-free survival [20]. However, high CKAP4 expression has also been described as indicating poor clinical prognosis [9,21]. There are also studies showing that CKAP4 is an anticancer protein despite being a protumor molecule. Further research into whether CKAP4 is a procancer or anticancer molecule are needed [18].

Similar to the protein DKK1, the CKAP4 levels in the present study were significantly higher in the patients with CRC than in the control group. Serum protein levels also increased in line with the grade and stage of the disease. However, serum CKAP4 levels gradually decreased following surgical resection.

A positive correlation was observed between the tumor diameter and protein levels. The decrease in protein levels following surgical resection explains the association between the tumor burden and serum levels. The two proteins have generally been examined separately in the literature, with tissue expression levels being investigated. The number of serum level studies is limited. The present study is the first to examine the levels of both proteins and has revealed a positive correlation between them.

This study compared the diagnostic efficacy of DKK1/CKAP4 with CEA and CA19-9, other biomarkers widely employed in CRCs and several other diseases. CEA is a tumor marker in gastrointestinal cancers, particularly CRC. It is used in the diagnosis, follow-up, and prognosis of CRC [22]. CEA and CA19-9 are monoclonal antibodies employed as serum markers for numerous diseases, including pancreatic, gastric, lung, biliary tract, colorectal, and other cancers [23,24]. Studies have shown that CEA exhibits sensitivity by 46–64% and specificity by 80–90%, while CA19-9 exhibits sensitivity by 14–34% and specificity by 55–89% in CRC [1,3,25]. Although these markers are routinely used in the diagnosis and follow-up of CRC, they do not yet possess sufficient diagnostic efficacy.

Although the diagnostic value of CEA and CA-19-9 in CRC was slightly higher than that in the literature in the present study, the diagnostic efficacy of DKK1/CKAP4 was above 95%. Furthermore, the diagnostic efficiency of CKAP4 was found to be higher than DKK1. Therefore, these proteins may represent biomarkers capable of use in both the diagnosis and follow-up of CRC. Although these proteins are currently expensive markers studied using the ELISA method today, their costs can be reduced with the help of autoanalyzers when other studies confirm their diagnostic efficacy.

This study has certain strengths and limitations. The first limitation is the relatively small number of patients due to the high cost of studying the markers. A second limitation is that due to budgetary constraints, the proteins’ tissue expression levels could not be investigated. Our study could not reveal how protein levels change in case of recurrence or progression of the disease. Another important limitation of our study is that it did not analyze how DKK1 and CKAP4 protein levels change over long periods, such as 6 months or 1 year. Lastly, examining the relationship between serum protein levels during the initial diagnosis and 1-, 3-, and 5-year survival outcomes can make an important contribution to the literature.

Despite these limitations, this study also has certain strengths. Previous studies have generally compared cancer patients with a healthy control group. In the present study, however, CRC patients were subdivided based on pathological grades and stages. One strength of this study is that serum protein levels were examined according to disease staging and grading. The fact that the two proteins were examined together and that correlations were measured may be another strength. Our next study is intended to perform immunohistochemical and molecular analyses on pathological specimens once an appropriate budget is made available, and to examine the expression level of proteins in tissues. One of our aims is to investigate the correlation between protein serum and tissue expression levels.

## 5. Conclusions

The DKK1 and CKAP4 serum values of patients with CRC are promising biomarkers. They are potential indicators in the diagnosis of CRC, treatment monitoring, and predicting tumor aggressiveness or disease severity. Future studies are needed to confirm these potential biomarkers. In addition, a reliable and low-cost test is necessary. In this way, it will be possible to apply DKK and CKAP4 in clinical practice. Further studies will be conducted to examine their serum and tissue expression levels to shed important light on this subject.

## Figures and Tables

**Table 1:** Demographic and clinicopathologic features of colorectal cancer patients.

Parameters	n
Age (years)	63.27 ± 9.7 (36–81)
Sex	
Female	23 (%)
Male	32 (%)
BMI (kg/m^2^)	24.91 (13.06–37.9)
Comorbidity	
Yes	28 (50.9)
No	27 (49.1)
COVID-19 history	15 (27.3%)
CEA (ng/mL) median (min–max) CA19-9 U/mL median (min–max)	4.1 (0.5–378) 14.2 (2–675)
Preoperative chemotherapy or radiotherapy	
Yes	18 (32.7%)
No	37 (67.3%)
Tumor location	
Right	12 (21.8%)
Left	22 (40%)
Rectum	21 (38.2%)
Operation type	
Laparoscopic	30 (54.5%)
Open	25 (45.5%)
pTNM Staging	
Stage I	11 (20%)
Stage II	18 (32.7%)
Stage III	21 (38.2%)
Stage IV	5 (9.1%)
Degree of diferentiation	
Poorly diferentiation (Grade I)	4 (7.3%)
Moderately diferentiation (Grade II)	43 (78.2)
Highly diferentiation (Grade III)	8 (14.5)
Tumor size (cm^3^)	46.45 ± 86 (0.6–504)
Postoperative complications within 30 days after surgery	18 (32.7%)

**Table 2:** DKK1–CKAP4 serum levels according to stages in colorectal cancer patients.

		n	Mean	Std. Deviation	Std. Error	Minimum	Maximum	*p*-Value
CKAP4	Stage I	11	794	196	59	556	1328	<0.05 *
	Stage II	18	1026	94	22	920	1320	
	Stage III	21	1270	158	34	1112	1796	
	Stage IV	5	1279	242	108	969	1589	
DKK1	Stage I	11	785	160	48	618	1200	<0.05 *
	Stage II	18	1051	58	13	947	1240	
	Stage III	21	1417	281	61	1133	2365	
	Stage IV	5	1524	558	249	1107	2472	

* Although no statistically significant difference was observed for either protein between stages 3 and 4 (*p* > 0.05), all the other stages differed significantly from one another (*p* < 0.05).

**Table 3:** Serum DKK1–CKAP4 levels according to grades in colorectal cancer patients.

		n	Mean	Std. Deviation	Minimum	Maximum	*p*-Value
CKAP4	Grade 1	4	806	211	556	1046	<0.05 *
	Grade 2	43	1100	226	652	1796	
	Grade 3	8	1216	250	767	1589	
DKK1	Grade 1	4	757	185	618	1025	<0.05 *
	Grade 2	43	1197	356	630	2472	
	Grade 3	8	1306	302	745	1802	

* Although no significant difference was observed for either protein between grades 2 and 3 (*p* > 0.05), all the other grades differed significantly (*p* < 0.05).

**Table 4:** CKAP4 and DKK1 levels of colorectal cancer and control group patients.

		n	Mean	SD	Minimum	Maximum	*p*-Value
CKAP4 (ng/L)	Preoperative period	55	1096	242	556	1796	<0.05 *
	POD 10	55	784	204	124	1477	
	POD 30	55	523	150	81	949	
	Healthy controls	40	187	68	93	427	
DKK1 (ng/mL)	Preoperative period	55	1181	358	613	2472	<0.05 *
	POD 10	55	833	270	328	1766	
	POD 30	55	531	207	183	1282	
	Healthy controls	40	215	108	104	652	

* There is a significant difference between all groups (one-way ANOVA test was applied).

**Table 5:** Correlation of DKK1-CKAP4 serum levels.

	Tumor Size	CKAP-4 Levels	DKK1 Levels
Correlation coefficient Tumor size Sig (2-tailed) n	1 - 52	0.356 ** 0.01 52	0.307 * 0.207 52
Correlation coefficient CKAP-4 levels Sig (2-tailed) n	0.356 ** 0.01 52	1 - 55	0.825 ** 0 55
Correlation coefficient DKK1 levels Sig (2-tailed) n	0.307 0.027 52	0.825 ** 0 55	1 - 55

Significant, powerful, positive correlations were observed between DKK1 and CKAP4 (r = 0.825, *p* < 0.001). Significant positive correlations were also determined between tumor diameter and DKK1 and CKAP4 levels (Table 5). * Low correlation; ** High correlation.

**Table 6:** ROC curve results and sensitivity and specificity values of colorectal cancer patients and healthy controls.

Parameters	Cut-Off Value	AUC (*p*-Value)	Sensitivity (%)	Specificity (%)
CKAP 4	743	0.998 (<0.001)	98	96
DKK1	811	0.994 (<0.001)	94	97
CEA	2.25	0.841 (<0.001)	77	74
CA 19-9	7.95	0.801 (<0.001)	75	87

## Data Availability

Data will be available upon reasonable request from the corresponding author.
